# Psychological and Psychosocial Effects of Cancer on Young Patients and Survivors in Italy: A Mixed-Methods Study on the Challenges and Meaning-Making Factors

**DOI:** 10.3390/diseases13110367

**Published:** 2025-11-10

**Authors:** Martina Gentile, Lucia Ronconi, Marco Romeo, Ciro De Vincenzo, Elena Burattini, Chiara Rutigliano, Nicola Santoro, Giulia Zucchetti, Franca Fagioli, Ines Testoni

**Affiliations:** 1 Department of Philosophy, Sociology, Education and Applied Psychology, University of Padua, 35139 Padua, Italy; martina.gentile@unipd.it (M.G.); ciro.devincenzo@unipd.it (C.D.V.); 2 Italian Associations of Volunteers Against Cancer (Anvolt), 60125 Ancona, Italy; semperonlus@gmail.com (M.R.); elena_burattini@libero.it (E.B.); 3 Computer and Statistical Services, Multifunctional Pole of Psychology, University of Padua, 35122 Padua, Italy; l.ronconi@unipd.it; 4 Semper ODV, Institute for Integrated Support and Multidisciplinary Research, 61032 Fano, Italy; 5 Pediatric Hemathology-Oncology Unit, University Hospital of Bari, 70124 Bari, Italy; rutigliano.chiara@gmail.com (C.R.); nico.santoro1956@libero.it (N.S.); 6 Paediatric Onco-Hematology, Stem Cell Transplantation and Cellular Therapy Division, Regina Margherita Children’s Hospital, 10126 Turin, Italy; giulia.zucchetti@unito.it (G.Z.); franca.fagioli@unito.it (F.F.); 7 Department of Paediatric and Public Health Sciences, University of Turin, 10126 Turin, Italy; 8 Department of Political Science, Law and International Studies, University of Padua, 35123 Padua, Italy

**Keywords:** pediatric cancer, psychological effects, young adults survivors, psycho-oncology

## Abstract

**Background:** Pediatric oncological diagnoses and treatments pose complex biopsychosocial challenges for both patients and their families. These experiences can significantly disrupt daily life, evoke intense emotional responses, and raise concerns about the future, often leading to long-term psychological implications. **Objective:** This study aimed to assess the emotional functioning of children undergoing cancer treatment and to explore the lasting psychological effects reported by young adult survivors of pediatric cancer. **Methods:** A mixed-methods approach was employed. In total, 52 minors and their caregivers were recruited from two pediatric oncology units in Italy (Turin and Bari), while 18 young adults diagnosed during childhood were recruited from clinical and community settings in Ancona and Bari. Standardized instruments were used to evaluate emotional symptoms and broader psychological functioning in the pediatric sample, while self-report measures assessed psychological well-being and quality of life in the young adult group. To complement these data, semi-structured interviews were conducted with the young adult survivors to investigate the long-term psychosocial and psychological impacts of pediatric cancer. **Results:** The quantitative results indicate generally low levels of psychological distress in both groups. However, the qualitative findings reveal the complexity of post-cancer identity, highlighting experiences of resilience, emotional ambivalence, and redefinition of self. **Conclusions:** These results underscore the importance of addressing the psychological well-being of pediatric oncology patients and young adult survivors beyond the medical treatment phase, with a focus on long-term psychosocial support and individualized care.

## 1. Introduction

Pediatric cancer presents a significant challenge not only from a medical perspective, but also due to the profound psychosocial implications it carries for affected children and their families. In Italy, approximately 1400 new cancer cases are diagnosed annually in children (ages 0–14) and around 600–800 in adolescents aged 15–20. Due to advances in treatment, over 80% of young patients now survive, with five-year survival rates nearing 85%. Leukemias and central nervous system tumors remain the most common forms across age groups [1].

Despite the significant progress in survival rates, the psychosocial burden associated with pediatric cancer remains considerable. Children frequently present with emotional and behavioral difficulties [2], while parents often experience heightened levels of anxiety and psychological distress, with family dynamics undergoing profound strain [3]. The repercussions extend beyond the period of active treatment: siblings may also develop emotional or behavioral problems, and parents are frequently required to balance demanding caregiving roles with work responsibilities, financial strain, and household management. In the absence of structured and sustained psycho-oncological support, the cancer experience may become traumatic during treatment and may continue to exert adverse psychological effects well beyond clinical remission [4]. Previous research has documented increased prevalence of anxiety, depression, social withdrawal, and post-traumatic stress symptoms in pediatric oncology [2,5,6], as well as significant caregiver burden and emotional exhaustion among parents [7,8,9,10].

Nevertheless, a comprehensive understanding of the long-term psychological consequences of pediatric cancer remains limited. In particular, little is known about the internal resources that contribute to resilience or about the psychological and psychosocial developmental trajectories of young adult survivors. Recent systematic studies confirm that anxiety, depression, and post-traumatic stress symptoms occur at elevated rates among pediatric oncology populations compared to healthy peers, highlighting the need for systematic psychosocial support [11,12]. Beyond documenting psychological risk, however, an increasing body of research emphasizes the importance of adaptive coping and resilience processes in shaping outcomes [13]. Phipps and colleagues (2015) have shown that many families demonstrate resilience in the face of pediatric cancer, with adaptive coping strategies buffering the impact of stressors on psychological adjustment [14]. Similarly, Rosenberg (2019) and collaborators have underscored the role of meaning-making interventions in promoting resilience and reducing distress among both patients and parents [15]. These findings converge with evidence that cognitive reappraisal, positive reframing, and social connectedness may operate as protective factors fostering benefit-finding and psychological growth [16]. Collectively, this evidence suggests that resilience in pediatric oncology is not merely the absence of psychopathology, but rather an adaptive process supported by coping resources and meaning-making capacities. This approach is critical during the transition from pediatric care into adulthood—a developmental phase often marked by complex challenges in identity formation, autonomy, and future planning. These domains may be profoundly influenced by the individual’s prior experience with cancer. Identity-related processes—such as whether survivors adopt a “survivor” or “victim” identity—have been found to influence mental health and quality of life in young adulthood [17]. Large-scale cohort data from the Dutch Childhood Cancer Survivor Study further indicate that, although adult survivors report levels of self-esteem and anxiety comparable to the general population, and even lower depression, they nevertheless experience poorer health-related quality of life and elevated rates of clinical distress [18]. Despite these advances, the number of studies that simultaneously integrate child self-reports and young adults survivors self report is still scarce, thus limiting the ability to design developmentally appropriate and family-centered psychosocial interventions.

To address these gaps, the present study investigates the psychological and psychosocial impact of pediatric cancer by combining quantitative data from both children undergoing treatment and their caregiver with both quantitative measures and insights qualitative insights from a separate sample of young adult survivors. This mixed-methods approach seeks to provide a deeper understanding of emotional experiences, psychological symptoms, and long-term consequences associated with pediatric cancer, with particular attention to the identification of personal resources and meaning-making processes that facilitate psychological adjustment and inform the development of comprehensive, evidence-based psychosocial care programs.

## 2. Materials and Methods

### 2.1. Study Design

This study adopted a mixed-method, cross-sectional design to examine the psychological and psychosocial impact of pediatric cancer across different phases of the illness trajectory. The research was structured around two primary objectives. The first aimed to evaluate the current psychological functioning of pediatric patients undergoing active treatment, using a quantitative approach based on data collected from both children and their caregivers. The second objective focused on investigating the long-term psychological effects of cancer in young adult survivors, employing a combination of standardized quantitative measures and semi-structured interviews. This dual approach allowed for a comprehensive analysis, integrating standardized psychological assessments with rich, narrative accounts of the lived experience of cancer survivorship. The study was approved by the Ethics Committee of the University of Padova (protocol code: 475-b).

### 2.2. Participants

The total sample consisted of 122 participants, divided into three subgroups in line with the study’s objectives: 52 minors, 52 caregivers, and 18 young adult survivors of pediatric cancer.

For the first objective, examining the psychological impact of pediatric cancer during the treatment phase, 52 minors (33 males, 19 females) and their respective caregivers (*n* = 52) were recruited from two pediatric hospitals: the Regina Margherita Pediatric Hospital in Turin (*n* = 24) and the Pediatric Unit of the Policlinico of Bari (*n* = 28). The mean age of the participating minors was 13.40 years (SD) with an age range of 6–17 years. Inclusion criteria for minors included an ongoing oncological diagnosis, an age up to 18 years, and the ability to participate in psychological assessments alongside a caregiver. Exclusion criteria comprised the presence of pre-existing neuropsychiatric disorders or language barriers that could compromise comprehension of the assessment tools. Regarding oncological diagnoses, the most frequent category was solid tumors (*n* = 18), followed by leukemias and myelodysplastic syndromes (*n* = 16), lymphomas (*n* = 10), and central nervous system tumors (*n* = 8). Sociodemographic characteristics of the participants are summarized in Table 1.

For the second objective, exploring the psychological and psychosocial long-term effects in individuals who had been diagnosed with cancer during childhood, 18 eligible participants (10 females and 8 males) were recruited from the Anvolt association headquarters in Ancona and the Pediatric Unit of the Policlinico of Bari. These participants had completed their treatment at least one year prior to enrollment and were able to reflect on their past oncological experiences from a post-treatment perspective. The mean age was 22 years (SD = 2.62).

The overall sample reflects a diversity of oncological diagnoses and family backgrounds, including individuals currently undergoing treatment as well as those who had transitioned into survivorship. Informed consent was obtained from all participants or, in the case of minors, from their parents prior to participation.

### 2.3. Outcome Measures

#### 2.3.1. Measures for Objective 1

To assess symptomatology in the first study, the following standardized questionnaires were administered from both self-reported and parent-reported perspectives:

**Children’s Depression Inventory 2–self report**

The Children’s Depression Inventory 2nd Edition (CDI 2:SR), Ref. [19], was used to assess cognitive, affective and behavioral signs of depression in children and adolescents. The 27 items are grouped into two scales: Emotional Problems and Functional Problems and four subscales (negative mood, interpersonal difficulties, ineffectiveness and negative self-esteem). The CDI 2 questionnaires hold license number 95606Z.

**Screen for Child Anxiety Related Disorders**

Screen for Child Anxiety Related Disorders (SCARED), Ref. [20] was used to assess children’s recent anxiety symptoms. It is a valid and sensitive measure to screen for pediatric anxiety disorders. Five subscales were examined: generalized anxiety symptoms, separation anxiety symptoms social anxiety symptoms, panic or somatic symptoms, and school avoidance. Authorization for its use was formally obtained from the Università Vita-Salute San Raffaele, Milan.

**Children’s Depression Inventory 2–parents**

The Children’s Depression Inventory 2nd Edition: Parent (CDI 2:P), Ref. [19] assesses depressive symptomatology in youth aged 7 to 17 years based on reports based on parental observations. This tool serves as a valuable complement to self-report measures, offering an external perspective on the emotional and behavioral functioning of youth.

**DSM 5-TR Self rated/Parent Cross-Cutting Symptom Measure (Levels 1 and 2)**

The Cross-Cutting Symptom Measure is structured into two levels. Level 1 provides a brief screening across 13 symptom domains for adults and 12 for children and adolescents, allowing for the identification of clinically relevant symptoms that may require further investigation. In this study, Level 1 assessments were administered to both children/adolescents (aged 11–17 years) and their parents to capture a wide range of psychiatric symptoms [21,22]. Subsequently, Level 2 evaluations focused on anger for the child/adolescent and anxiety for the parent/caregiver. This tiered approach facilitated a nuanced understanding of symptomatology from both self- and caregiver-reported perspectives [23,24]. The instruments were accessed from the official website of the publisher, where they are freely available for research and clinical use.

#### 2.3.2. Measures for Objective 2

In the second study, the following validated instruments were employed to investigate the psychosocial impact of the illness experience, with particular attention to quality of life, emotional well-being, and perceived changes in daily functioning through semi-structured interviews:

**Beck Depression Inventory II**

Beck Depression Inventory II (BDI II), Ref. [25] is a valid tool for the measurement of major depression symptoms according to diagnostic criteria listed in the Diagnostic and Statistical Manual for Mental Disorders.

**The World Health Organization Quality of Life–BREF**

The WHOQOL-BREF (WHOQOL), Ref. [26] is a self-report questionnaire designed to assess an individual’s perceptions of their quality of life, including aspects related to their health and overall well-being. It consists of 4 domains of quality of life: physical health, psychological health, social relationships, and environment. In addition, there are 2 items that measure overall QOL and general health. The instrument was obtained from the official repository of the World Health Organization, where it is freely available for research and educational purposes.

**Semi-structured Interview**

The semi-structured protocol was developed following a thorough review of the scientific literature [27,28] in order to ensure the inclusion of the most relevant themes within the field. It was designed to explore key dimensions of the participants’ lived experiences, allowing for both consistency across interviews and flexibility to probe individual narratives in greater depth. Grounded in a constructivist framework, the protocol emphasized the co-construction of meaning during the interview process and the exploration of participants’ subjective perceptions and sense-making. The main domains addressed included the impact of illness on daily life, changes in social relationships, the psychological effects of medical treatments, the perception of potential positive aspects arising from the illness experience, and reflections on future goals, including educational and career aspirations. Some questions from the protocol: “In what ways do you feel the medical treatment has affected your everyday life? What aspect of this impact has been the most substantial or meaningful to you?”, “Has the illness influenced your aspirations or plans for the future? If so, in what ways?”.

This approach aimed to elicit rich, nuanced accounts while maintaining alignment with established research priorities.

### 2.4. Data Analysis Techniques

#### 2.4.1. Quantitative Analysis

Quantitative analyses were performed to examine the strength and direction of associations between self-report and parent-report measures. Pearson correlation coefficients were computed to assess the degree of linear relationship between variables. The interpretation of correlation magnitudes followed Cohen’s (1988) guidelines [29]: correlations below 0.1 were considered very small effects, values between 0.1 and 0.3 small effects, between 0.3 and 0.5 moderate effects, and correlations equal to or above 0.5 were interpreted as large effects. Statistical significance was set at *p* < 0.05, with levels of significance denoted by asterisks (* *p* < 0.05; ** *p* < 0.01).

To evaluate agreement between parent and child reports on categorical symptom severity scales, cross-tabulations and Kappa statistics were conducted. The Kappa coefficient was used to quantify the level of agreement beyond chance, with statistical significance determined at *p* < 0.05. Descriptive statistics including means and standard deviations were calculated to compare symptom severity levels reported by parents and children.

#### 2.4.2. Qualitative Analysis

This study adopted a qualitative, cross-sectional design, employing Reflexive Thematic Analysis (RTA), Ref. [30] to systematically identify and interpret recurring themes within the narrative data. RTA provides a critical and reflexive framework that emphasizes the recursive and iterative nature of the analytical process, allowing for an integration of both theory-driven and data-driven insights through an abductive approach. The analysis was conducted using Atlas.ti 2025 [31], which supported the efficient organization and management of data. To further strengthen the transparency and rigor of the analytic procedure, we systematically tracked the evolution of codes across different phases of the analysis. This approach not only enhanced accountability, but also offered the research team clear signposts and waypoints to revisit if certain coding strategies proved unproductive. Specifically, we highlighted all changes made to codes during each successive iteration. A Microsoft Excel (2016) spreadsheet was established to document the coding trajectory and decisions [32]. Table 2 provides an excerpt from the spreadsheet used to track the coding process. This table includes codes developed during the first iteration exported directly from Atlas.ti, each linked to the corresponding participant and quotation. Subsequent coding iterations were systematically recorded in the same file, offering a comprehensive overview of the evolution and refinement across the different phases of analysis. The coded data are examined and analyzed to determine how different codes can be grouped based on shared meanings, allowing for the development of themes or sub-themes.

## 3. Results

### 3.1. Quantitative Results

#### 3.1.1. Descriptive Results

In the first study, we observed the following descriptive results. These findings are comprehensively summarized in an aggregated table for enhanced clarity and readability (Table 3).

The distribution of scores on the Children’s Depression Inventory-II, (CDI-II) indicated that the majority of participants (82.7%, *n* = 43) fell within the medium or low symptom severity categories. Smaller proportions were classified as average (7.7%, *n* = 4) or high (7.7%, *n* = 4), while only a minimal percentage (1.9%, *n* = 1) were categorized as very high. When examining the Emotional Problems subscale, 86.5% of the sample (*n* = 45) were in the medium or low range, 11.5% (*n* = 6) fell into the above-average category, and 1.9% (*n* = 1) were classified as high. For the Functional Problems subscale, 71.2% (*n* = 37) of participants scored in the medium or low range, 13.5% (*n* = 7) were above average, and both the high and very high categories comprised 7.7% each (*n* = 4). Domains assessed through the DSM-5 Level 1 measures revealed predominantly low symptom expression: somatic symptoms were absent or very mild in 63.2% of participants, sleep disturbances in 68.4%, attention difficulties in over 89%, depressive symptoms in 63.2%, and anger, irritability, mania, and anxiety symptoms in approximately 60–70%. Psychotic symptoms were rarely reported (89.5% absent), and repetitive thoughts or behaviors were absent in 52.6% of participants, with only 5.3% reporting severe symptoms. Similarly, on the DSM-5 Level 2 Cross-Cutting Symptom Measure–Anger, 86.8% of participants reported absent or sporadic symptoms, with only a minority indicating mild (2.6%) or moderate (10.5%) anger. According to the Screen for Child Anxiety Related Emotional Disorders (SCARED), 68.6% of the sample scored within the normal range, while 31.4% met the threshold for anxiety disorder.

Parent-reported data largely mirrored these findings. On the DSM-5 Level 1 Cross-Cutting Symptom Measure, symptoms across most domains—including anxiety, somatic complaints, sleep disturbances, inattention, and depression—were absent or mild in the majority of cases. Irritability, mania, psychosis, repetitive behaviors, substance use, and suicidal ideation were infrequent. Parent ratings on the DSM-5 Level 2 Anxiety Scale showed 72.1% of participants with anxiety rated as absent or sporadic. On the parent version of the CDI-II, 73.5% scored in the medium or low range for depressive symptoms and 81.6% for functional problems, while emotional problems showed greater variability, with fewer than half falling within the medium or low range.

In the second study, among the 18 participants, the majority (72.2%) exhibited mild depressive symptoms, followed by 22.2% with moderate symptoms, and 5.6% with severe depression, assessed by BDI-II. Descriptive statistics from the WHOQOL-BREF indicated that the highest mean score was found in the Physical Health domain (M = 73.81, SD = 17.88), followed by the Environment domain (M = 69.25, SD = 14.40), Social Relationships (M = 64.81, SD = 25.49), and Psychological well-being (M = 62.50, SD = 19.65). Participants rated their overall quality of life with a mean score of 66.67 (SD = 21.00), while satisfaction with their own health had a slightly lower mean of 63.89 (SD = 23.04). The results are presented in aggregated table (Table 4).

#### 3.1.2. Correlations

Among the observed associations, the three strongest and statistically significant correlations were found between the DSM-5 Level 2 Cross-Cutting Symptom Measure Anger/Irritability (self-report) and the corresponding parent-report version (r = 0.604, *p* < 0.01), the DSM-5 Level 1 Cross-Cutting Symptom Measure Somatic Symptoms (self-report) and the corresponding parent-report version (r = 0.558, *p* < 0.01), and the DSM-5 Level 1 Cross-Cutting Symptom Measure Sleep Disturbances (self-report) and its parent-report counterpart (r = 0.543, *p* < 0.01).

To further examine the level of agreement between parent and child reports of depressive symptoms, paired samples correlations and mean comparisons were conducted using the CDI-2 Total Score, Emotional Problems, and Functional Problems subscales. Results indicated moderate and statistically significant correlations between parent and child reports for the Total Score (r = 0.445, *p* < 0.001) and the Emotional Problems subscale (r = 0.427, *p* = 0.002). In contrast, the correlation for the Functional Problems subscale did not reach statistical significance (r = 0.231, *p* = 0.132). Paired t-test results revealed that parents systematically reported higher levels of depressive symptoms than their children (t = 4.36 df = 48 *p* < 0.001 for the total score and t = 7.18 df = 48 *p* < 0.001 for the total score). Specifically, the mean Total Score was higher in the parent report (M = 55.92, SD = 9.36) compared to the self-report (M = 50.10, SD = 8.27). A similar pattern emerged for the Emotional Problems subscale, with parents reporting higher scores (M = 60.04, SD = 10.47) than children (M = 49.86, SD = 7.45).

The cross-tabulation between parent- and child-reported sleep disturbances (Level 1 Cross-Cutting Symptom Measure) revealed a modest level of agreement. While 50% of parents rated their child as having no sleep disturbances, only 27.8% of children reported the same. When combining the “Very mild” and “Mild” categories, discrepancies became evident; notably, some children who self-reported moderate to severe sleep problems were rated by their parents as having no or only mild symptoms.

Similarly, the contingency table comparing somatic symptom severity between self-report (Somatic symptoms) and parent-report (Somatic symptoms) versions of the Cross-Cutting Symptom Measure showed moderate concordance. Among 36 participants, symptom severity categories overlapped particularly in the “Mild” and “Moderate” ranges. A considerable proportion of cases classified as “Mild” or “Moderate” by self-report were also rated similarly by parents, although some discrepancies were present across severity levels. The Kappa statistic, which quantifies agreement beyond chance, was 0.396 (SE = 0.099), indicating fair to moderate agreement between self- and parent-reports, and this result was statistically significant (*p* < 0.001).

Likewise, the contingency analysis of anger and irritability symptoms between self-report and parent-report measures demonstrated moderate overlap across severity categories in the same sample of 36 participants. The highest frequencies were observed in the “Mild” category for both informants, with 47.2% of cases classified as mild by parent-report and 38.9% by self-report. However, some variation in ratings was observed, with parents tending to assign slightly higher proportions to the “Absent” or “Very Mild” categories compared to children’s self-reports

### 3.2. Qualitative Results

The qualitative analysis yielded three overarching themes that illuminate the psychological and experiential dimensions of survivorship. The first theme, Suffering as an Epistemic Experience, highlights how illness reshaped participants’ values and sense of self. The second, Two-Timed Healing: The Discrepancy Between Bodily Recovery and Self-Integration, captures the discrepancy between physical healing and intrapersonal adaptation. The third, Between Vulnerability and Enforced Becoming, illustrates how survivors navigated both heightened fragility and premature responsibilities.

These themes are detailed below and illustrated with representative quotations, with a summary provided in Table 5.

Theme 1: Suffering as an Epistemic Experience

Participants’ accounts revealed that psychophysical suffering resulting from cancer was not solely perceived as a source of distress, but also as a transformative experience that profoundly reshaped their understanding of life and Self. This process appeared to initiate a reconfiguration of values and priorities, a verticalization of the value system, which marked a clear boundary between the self and others who had not experienced illness.

Subtheme 1: Radical transcendence of life priorities

The experience of illness was frequently narrated as a catalyst for personal growth and reevaluation of what truly matters in life. Many participants described a deepened awareness of the value of relationships, daily experiences, and moments often taken for granted prior to the diagnosis, for instance one states: “It makes you aware of your own life, the meaning of small things, relationships, the luck of having friends, family, and so on”. This reorientation often involved a shift in focus from externally imposed expectations to internally derived meaning. For example, another accounts: “I’m happy I went through it, because it made me discover so many positive aspects of life… it helps you realize what’s really important”. Another interviewed added: “Sometimes I say it was a positive experience, because now I can understand so much more… you really learn what matters, you re-evaluate your priorities.”

Subtheme 2: Knowledge That Separates from the Other

Participants described how this transformation in perspective often led to feelings of disconnection from peers who had not undergone a similar experience. The illness appeared to draw an invisible line between those who had faced life-threatening conditions and those who continued to engage with life’s ordinary concerns. According to one: “Problems that seem insurmountable to others are simple for me”. Participants reported difficulty relating to their peers’ preoccupations, which now seemed superficial or misaligned with their new worldview. In the words of a participant: “You realize that priorities are completely different… I would watch some of my friends getting upset because boys didn’t like them”.

Theme 2: Two-Timed Healing: The Discrepancy Between Bodily Recovery and Self-Integration.

While healing can be measured in clinical terms within oncology, psychological time follows a different logic. The process of accepting change and reconstructing the self cannot be anticipated or quantified. It unfolds gradually and requires an additional, often overlooked, phase of care aimed at supporting emotional and existential integration beyond physical recovery.

Subtheme 1: Euphoric phase at Treatment Completion

Many participants described an initial phase of euphoria following the end of treatment, experienced as a moment of liberation and the reclaiming of freedom and autonomy, as illustrated by one account: “I remember the first period was very euphoric. The joy of being able to do everything again made me feel like I was constantly in motion, never stopping.” However, this phase appears fragile and transient, an emotional “bubble” that conceals the complexity of the inner experience, like one who: “During the first year, I think I was caught in this bubble of excitement, the thrill of being able to do everything again. But I was setting aside many other things”.

Subtheme 2: Occupying a liminal emotional space

While the body may be clinically “free” of disease, the emotional experience remains deeply shaped by the oncological journey. The return to regularity is not immediate, but is instead marked by anxiety, fear, and a persistent sense of uncertainty. As one reported: “Every step I took seemed to depend on that fear [of recurrence].” Or another: “In the period afterwards, I couldn’t go anywhere without checking if there was a hospital nearby.” Another described this feeling with the following metaphor: “I felt like I had one foot on each side”. These narratives reveal a lasting internalization of the illness experience, which continues to influence daily behaviors, desires, and future planning. The past intrudes upon the present, making it difficult to envision the future: “I couldn’t bring myself to desire.” Yet, some participants are able to name a kind of “healthy fear,” recognizing vulnerability as part of the process of reclaiming one’s sense of self. “Fear, yes, but a healthy fear… it was like taking the first steps on something new”.

Theme 3: Between Vulnerability and Enforced Becoming

Healing unfolds within a body and mind marked by the illness where cancer has disrupted internal balance and redrawn boundaries. From this altered ground, participants described a pressing need to re-establish stability, often through a premature engagement with adult concerns such as work, responsibility, and future planning. Vulnerability and forced maturation coexist, shaping a sense of self caught between recovery and becoming.

Subtheme 1: Psychocorporeal Vulnerability and Identity Challenges after Treatment Completion

The accelerated maturation triggered by illness does not always culminate in a stable sense of self. For many individuals, vulnerability resurfaces or even intensifies following the completion of treatment. Manifestations such as eating disorders, health-related anxiety, and identity crises reflect the difficulty of managing the burden imposed by a developmental acceleration that is incongruent with the social environment or personal emotional readiness. One participant shared, “Psychologically, I felt really unwell after treatment… the first year I was maybe in this bubble of enthusiasm… but then I became very scared.” Another reported, “When I finished treatment, I started losing weight… that eventually led to an eating disorder.” A further account described, “I had extreme hypochondria… I used to film myself taking my medications because a minute later I wouldn’t remember.” Additionally, one participant noted, “Whenever I left home for a trip, I had to make sure there were hospitals nearby.”

Subtheme 2: Early evolutionary stages

Participants described illness as an event that forces a premature transition into adulthood. The diagnosis interrupts the temporal continuity of development and introduces decision-making, and responsibilities. Some young individuals were required to make fertility preservation decisions, such as oocyte cryopreservation, as a young adult stated: “I froze my ova. It wasn’t an easy process with the hormones, but it had to be done, so I did it”. Existential uncertainty brought on by illness appears to foster a strong desire for financial security and personal autonomy, as one reported: “The first thing you do is get a stable job… maybe not start a family right away, but at least, yes, secure a very stable future earlier than most people probably do”.

## 4. Discussion

This study investigated the psychological functioning of pediatric oncology patients and young adult survivors, capturing both the acute experience of illness and the long-term consequences of survivorship. By integrating quantitative and qualitative data, we aimed to provide a comprehensive understanding of the emotional and developmental impact of cancer across different life stages.

Quantitative findings indicate generally low-to-moderate levels of psychological symptomatology in both children undergoing active treatment and young adult survivors. In the pediatric sample, symptoms of depression and anxiety were predominantly reported in the low range, consistent with prior research highlighting the resilience and adaptive functioning often observed in children with cancer [33,34,35]. Recent systematic reviews further confirm that, although rates of anxiety and depression are elevated in pediatric oncology populations compared to healthy peers, most patients exhibit subclinical levels of distress, suggesting considerable adaptive capacity in the face of illness [11,12]. This pattern of adaptive coping indicates that, despite the challenges posed by treatment and hospitalization, many pediatric patients are able to preserve psychological well-being, likely supported by protective factors such as stable family environments, access to psychosocial care, and individual strengths like optimism and effective coping strategies.

This adaptive functioning can be understood through resilience frameworks proposed by Phipps and colleagues, which conceptualize resilience not as the absence of distress, but rather as the ability to sustain or regain emotional balance through mechanisms such as positive reappraisal, meaning making, and benefit finding [14]. Similarly, Willard and Tillery emphasize the role of coping, flexibility, hope, and cognitive reframing as key predictors of psychological adjustment and quality of life among young survivors [36]. From this perspective, our findings of low-to-moderate distress may reflect the engagement of adaptive coping processes rather than an absence of emotional challenge.

An important discrepancy emerged, however, between children’s self-reports and parent-proxy reports, with caregivers consistently perceiving higher levels of depressive symptoms than their children. This finding reflects a broader trend of low-to-moderate concordance in parent–child symptom ratings, particularly in internalizing domains such as emotional symptoms and physical symptoms [37,38,39]. While such discrepancies may stem from heightened parental vigilance or concern, they also introduce relevant clinical implications. Excessive parental worry, when left unaddressed, may inadvertently reinforce a sense of vulnerability in the child, compromising emotional security and autonomy [40]. Interventions focusing on parental stress management and resilience-promoting skills reduce caregiver anxiety and post-traumatic stress symptoms, suggesting that addressing parental distress can foster a more secure and supportive environment for the child [15]. Interestingly, in our study, the levels of agreement between children and parents were observed in domains such as anger and irritability, somatic complaints, and sleep disturbances. Notably, the strong parent–child agreement regarding sleep disturbances highlights an area of clinical priority, as sleep plays a fundamental role in children’s neurocognitive, emotional, and physical development [41]. Moreover, the bidirectional relationship between pain and sleep, where pain disrupts sleep and disturbed sleep, in turn, intensifies psychological symptoms and increases pain sensitivity, as Valrie [42] demonstrated, adds further urgency to addressing these symptoms. Within this cycle, irritability may serve as an intermediary factor, intensifying emotional strain and contributing to greater functional impairment.

The adaptive profile emerging from the quantitative findings was further deepened by qualitative data collected from young adult survivors. Young adult survivors retrospectively described their illness not only as a disruptive life event, but also as a transformative experience that led to greater self-awareness, a redefinition of life priorities, and an increased appreciation for meaningful relationships and everyday moments. These narratives align with the framework of post-traumatic growth [43], suggesting that the resilience developed during treatment may serve as a foundation for longer-term psychological reconstruction. At the same time, participants emphasized that such growth did not occur in isolation from distress. Post-traumatic growth frequently coexisted with latent anxieties, fear of recurrence, and a fragile sense of stability. This ambivalence indicates that resilience is not a linear pathway toward well-being, but a dynamic process in which adaptive resources and enduring vulnerabilities remain intertwined. Young adult survivors often articulated this tension when reflecting on the idea of being “free” from cancer, which they experienced as both empowering and anxiety-provoking. This tension underscores the complexity of post-cancer identity formation, especially during adolescence and young adulthood, developmental periods already characterized by intense identity exploration [44].

The transition from active treatment to survivorship was described as particularly disorienting. While this phase is typically defined by clinicians as a period of recovery, young adult survivors often experienced it as a liminal space marked by persistent fear of recurrence, confusion about identity, and a fragile sense of stability. Metaphors such as “occupying a liminal state” reflected this psychological in-betweenness. These accounts suggest that survivorship should not be conceptualized as a static outcome, but rather as a dynamic and ongoing process of adaptation. Structured psychological support during this phase may be crucial in addressing the latent forms of distress that emerge when the immediate demands of treatment subside [2,45].

Moreover, many young adult survivors reported feeling pressured to make accelerated life decisions particularly concerning career trajectories and reproductive planning, often in the absence of adequate developmental readiness. While some individuals perceived these challenges as opportunities for personal growth and increased autonomy, others described them as premature burdens that could elicit a sense of dissonance between their chronological age and perceived psychosocial maturity. This phenomenon, often articulated as feeling “older than one’s peers,” highlights the experience of forced or accelerated adulthood.

This suggests the need for developmentally sensitive follow-up care that not only monitors medical outcomes, but also supports the evolving psychosocial needs of young adult survivors as they navigate young adulthood.

## 5. Conclusions

This study offers a nuanced portrait of the psychological impact of pediatric cancer across treatment and survivorship; however, several limitations warrant consideration. First, the small sample size of young adult survivors (*n* = 18) limits the generalizability of the findings and reduces the statistical power of the correlational analyses. To address this limitation and enhance the interpretative richness of the study, a mixed-methods approach was intentionally adopted, allowing for the integration of qualitative insights to complement and contextualize the quantitative data.

Second, the cross-sectional design of this study limits our ability to interpret how psychological and emotional processes evolve over time. While valuable in capturing the perspectives of the participants’ experiences, this methodology does not allow for tracking changes in psychological functioning, coping strategies, or meaning-making across different stages of the illness and survivorship. Longitudinal studies would be better suited to assess these dynamic processes and to identify potential turning points in psychological adaptation. However, these differences should not be seen solely as methodological limitations, but rather as clinically informative data points. Discrepancies between child and parent perceptions can serve as valuable entry points for clinical intervention, helping practitioners explore how each informant is interpreting the symptom, whether they are responding to observable behaviors, internal emotional states, or potentially influenced by cognitive or emotional biases. Understanding the nature of disagreement may enhance diagnostic accuracy and inform more personalized interventions.

Another limitation concerns the use of different assessment tools for children and caregivers within our sample may have contributed to variation in reported symptoms and limited direct comparison. Future studies would benefit from employing parallel or identical instruments across informants to enable more precise analyses of inter-rater agreement. Finally, although this study extensively discusses resilience and adaptive coping, no specific instruments specifically designed to assess resilience, post-traumatic growth, or treatment-related were employed. This represents a limitation, as such measures could have provided more direct insights into the ways in which oncological treatment and survivorship trajectories shape psychological outcomes. As highlighted by Marbot [46], several validated tools exist for measuring psychological resilience in childhood cancer survivors and their families (CD-RISC, 12 Resilience Scale for Adults, 18, 19 and BRS2), and future research should also consider.

Clinically, our findings support the development of family-centered interventions that target observable domains such as irritability, somatic complaints, and sleep disturbances—areas in which parent–child agreement was strongest. These results also emphasize the need for developmentally sensitive care that attends to both symptom management and the evolving identity challenges that persist into survivorship. Finally, longitudinal, multi-informant research is needed to clarify the dynamics of psychological adaptation over time, the role of caregiver perception, and the effectiveness of tailored interventions that support the entire family system in navigating life after pediatric cancer.

## Figures and Tables

**Table 1:** Socio-demographics of each sub sample.

Characteristics	Minors	Caregivers	Young Adult Survivors	
	(*n* = 52)	(*n* = 52)	(*n* = 18)	
	n (%) or mean (SD)	n (%) or mean (SD)	n (%) or mean (SD)	
Origin				
	Anvolt association	/	/	18 (100%)
	Pediatric Unit of hospital in Bari	28 (54%)	28 (54%)	/
	Pediatric hospital in Torino	24 (46%)	24 (46%)	/
Age		13.4 (3.82)	44.1 (5.98)	22.2 (2.62)
Gender				
	Female	19 (36%)	39 (75%)	10 (56%)
	Man	33 (64%)	13 (25%)	8 (44%)
Educational level				
	Low	/	16 (31%)	0 (0%)
	Secondary school or above	/	35 (67%)	18 (100%)
	Missing	/	1 (2%)	

**Table 2:** Excerpt of spreadsheet tracking code changes.

Participant	Quotation Content	Iteration 1	Iteration 2
**1**	I don’t know how I can evaluate it. There’s been a lot of change.	She does not know how to evaluate it, but reports that “there has been a lot of change”.	Implicit and non-verbalizable transformation
**2**	After the illness, I looked for different kinds of connections.	He sought different kinds of social connections after the illness	Emergence of new relational needs
**3**	Sometimes I even say that, in a way, it was a positive experience for me, because now I’m able to understand many things about how the world works. It helps you realize what really matters in life	She reports a deeper understanding of how the world works	Perceived acquisition of existential insight
**10**	At the beginning, the illness limited me a lot. I was very young—I was in the first year of middle school. So, finding myself suddenly thrown into all of this was difficult. I was already having trouble with my class, because I was often the one who got bullied the most—for my slightly crossed eye, for being a bit more overweight, for being shorter than the others	Early bullying due to physical changes caused by the illness	The embodied weight of being seen through illness
**11**	I realize that maybe I’m much more mature than other people—I grew up faster	Growth and maturation ahead of peers	The lived experience of growing older inside before one’s time

**Table 3:** Descriptive statistics of self and parent reported measures in pediatric patients.

Measure		
	Category/Subscale	N Percentage (%)
CDI-II	Medium or Low	82.7
Total score	Average	7.7
	High	7.7
	Very High	1.9
	Medium or Low	86.5
Emotional Problems	Above Average	11.5
	High	1.9
	Medium or Low	71.2
	Above Average	13.5
Functional Problems	High	7.7
	Very High	7.7
	Absent or very mild	63.2
	Absent or very mild	68.4
DSM-5	Absent or very mild	>89
Somatic Symptoms	Absent or very mild	63.2
	Absent or very mild	60–70
	Absent	89.5
	Absent	52.6
Sleep Disturbances	Severe	5.3
	Absent or sporadic	86.8
	Mild	2.6
	Moderate	10.5
Attention Difficulties	Normal range	68.6
	Threshold for anxiety disorder	31.4
	Anxiety, Somatic, Sleep, Inattention, Depression	Absent or mild in the majority of cases
	Irritability, Mania, Psychosis, Repetitive Behaviors, Substance Use, Suicidal Ideation	Infrequent
Depressive Symptoms	Absent or sporadic	72.1
	Depressive Symptoms (Medium or Low)	73.5
	Functional Problems (Medium or Low)	81.6
	Emotional Problems (Medium or Low)	<50

**Table 4:** Descriptive statistics of self-report in young adults survivors.

Measure			
BDI-II	Category/Subscale	N Percentage (%)	Mean Score(M)
Total score	Mild depressive symptoms	72.2	–
Total score level	Moderate depressive symptoms	22.2	–
	Severe depression	5.6	–
	Physical Health	–	73.81
	Environment	–	69.25
WHOQOL-BREF	Social Relationships	–	64.81
Physical Health	Psychological well-being	–	62.50
Environment	Overall quality of life	–	66.67
Social Relationships	Satisfaction with own health	–	63.89

**Table 5:** The three themes from reflexive thematic analysis and examples of quotations.

Themes	Subthemes	Example Quote
Suffering as an Epistemic Experience	Radical transcendence of life priorities	It makes you aware of your own life, the meaning of small things, relationships, the luck of having friends, family, and so on
	Knowledge That Separates from the Other	Other people worry about things that I think are not a big deal
Two-Timed Healing: The Discrepancy Between Bodily Recovery and Self-Integration	Euphoric phase at Treatment Completion	During the first year, I think I was caught in this bubble of excitement, the thrill of being able to do everything again. But I was setting aside many other things
	Occupying a liminal emotional space	I felt like I had one foot on each side
Between Vulnerability and Enforced Becoming	Psychocorporeal Vulnerability and Identity Challenges after Treatment Completion	When I finished treatment, I started losing weight that eventually led to an eating disorder
	Early evolutionary stages	The first thing you do is get a stable job maybe not start a family right away, but at least, yes, secure a very stable future earlier than most people probably do

## Data Availability

The data presented in this study are available on request from the corresponding author. The data are not publicly available due to privacy.
